# *Six3* demarcates the anterior-most developing brain region in bilaterian animals

**DOI:** 10.1186/2041-9139-1-14

**Published:** 2010-12-29

**Authors:** Patrick RH Steinmetz, Rolf Urbach, Nico Posnien, Joakim Eriksson, Roman P Kostyuchenko, Carlo Brena, Keren Guy, Michael Akam, Gregor Bucher, Detlev Arendt

**Affiliations:** 1 Developmental Biology Unit, European Molecular Biology Laboratory, Meyerhofstrasse 1, 69012 Heidelberg, Germany; 2 Johannes Gutenberg-Universität Mainz, Institut für Genetik, J.-J.-Becher-Weg 32, 55128 Mainz, Germany; 3 Johann-Friedrich-Blumenbach-Institute of Zoology, Anthropology and Developmental Biology, DFG Research Centre for Molecular Physiology of the Brain (CMPB), Georg August University, von-Liebig-Weg-11, 37077 Göttingen, Germany; 4 University Museum of Zoology, Department of Zoology, Downing Street, Cambridge CB2 3EJ, UK; 5 Department of Embryology, State University of St. Petersburg, Universitetskaya nab. 7/9, 199034 St. Petersburg, Russia; 6 University of Vienna, Department for Molecular Evolution and Development, Althanstrasse 14, A-1090 Vienna, Austria; 7 Vetmeduni Vienna, Institute of Population Genetics, Veterinärplatz 1, A-1210 Vienna, Austria; 8 Queen Mary University of London, School of Biological and Chemical Sciences, Mile End Road, London E1 4NS, UK

## Abstract

**Background:**

The heads of annelids (earthworms, polychaetes, and others) and arthropods (insects, myriapods, spiders, and others) and the arthropod-related onychophorans (velvet worms) show similar brain architecture and for this reason have long been considered homologous. However, this view is challenged by the 'new phylogeny' placing arthropods and annelids into distinct superphyla, Ecdysozoa and Lophotrochozoa, together with many other phyla lacking elaborate heads or brains. To compare the organisation of annelid and arthropod heads and brains at the molecular level, we investigated head regionalisation genes in various groups. Regionalisation genes subdivide developing animals into molecular regions and can be used to align head regions between remote animal phyla.

**Results:**

We find that in the marine annelid *Platynereis dumerilii*, expression of the homeobox gene *six3 *defines the apical region of the larval body, peripherally overlapping the equatorial *otx+ *expression. The *six3+ *and *otx+ *regions thus define the developing head in anterior-to-posterior sequence. In another annelid, the earthworm *Pristina*, as well as in the onychophoran *Euperipatoides*, the centipede *Strigamia *and the insects *Tribolium *and *Drosophila*, a *six3/optix+ *region likewise demarcates the tip of the developing animal, followed by a more posterior *otx/otd+ *region. Identification of *six3*+ head neuroectoderm in *Drosophila *reveals that this region gives rise to median neurosecretory brain parts, as is also the case in annelids. In insects, onychophorans and *Platynereis*, the *otx+ *region instead harbours the eye anlagen, which thus occupy a more posterior position.

**Conclusions:**

These observations indicate that the annelid, onychophoran and arthropod head develops from a conserved anterior-posterior sequence of *six3+ *and *otx+ *regions. The *six3+ *anterior pole of the arthropod head and brain accordingly lies in an anterior-median embryonic region and, in consequence, the optic lobes do not represent the tip of the neuraxis. These results support the hypothesis that the last common ancestor of annelids and arthropods already possessed neurosecretory centres in the most anterior region of the brain. In light of its broad evolutionary conservation in protostomes and, as previously shown, in deuterostomes, the *six3*-*otx *head patterning system may be universal to bilaterian animals.

## Background

**Figure 1:**
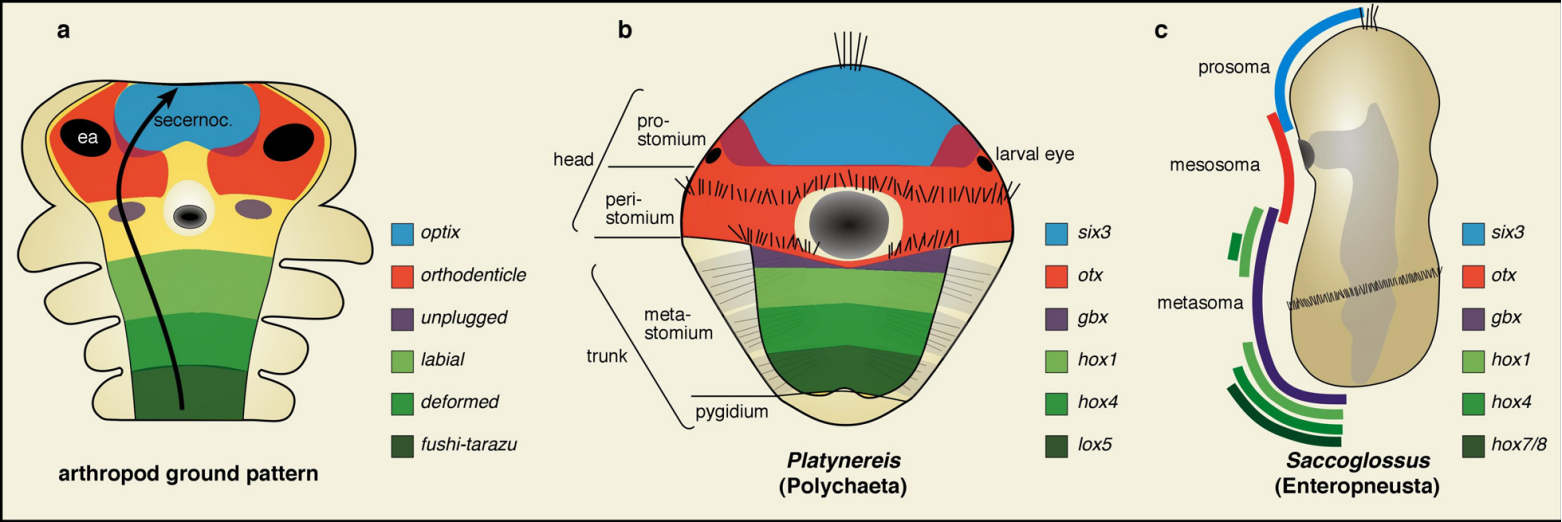
**Conservation of anterior-posterior *six3*/*optix-, gbx*/*unplugged *and Hox-expressing territories in Bilateria**. A conserved anterior-posterior alignment of *six3*/*optix*-, *otx*/*otd*-, *gbx*/*unplugged*- and *hox*-expressing neuroectodermal regions in the hypothetical ancestral arthropod (a), the annelid *Platynereis *(b), and the hemichordate *Saccoglossus *(c). (a) Arrow depicts the antero-posterior neuraxis pointing at the anterior-most *six3*/*optix*-region as identified by the data presented here. Light grey in (b): developing parapodial appendages, in (c): gut. Dark grey: mouth opening. Yellow: neuroectoderm not expressing any of the mentioned genes. Purple in (a, b): *six3+*/*otx+ *regions. All animals are oriented with anterior to the top. (a, b): ventral views. (c): lateral view. ea: eye anlage. Arthropod schematic after [29,36-38,48-53], *Platynereis *and *Saccoglossus *schematics after references in the text.

The brains of annelids and arthropods are similarly composed of cerebral ganglia located above the foregut and a variable number of associated segmental ganglia, incorporated to the brain through cephalisation [1,2]. In annelids, the cerebral ganglia develop, at least in their largest part, from the neuroectoderm of the prostomium, the most anterior part of the annelid body. In polychaete annelids with indirect development, the prostomium forms from the larval episphere, the upper half of the trochophora larva (the apical "cap" anterior to the primary trochoblasts forming the prototroch ciliary ring) (Figure 1b). A smaller subset of cerebral neurons forms from the peristomium, the more posterior part of the developing head that contains the mouth and lies anterior to the first metameric segment. The peristomium forms from the equatorial larval regions including the larval foregut (stomodaeum), the prototroch and metatroch ciliary bands if present (Figure 1b) [3,4].

In arthropods, the cerebral ganglia are composed of the protocerebrum and two segmental neuromeres, the deuto- and tritocerebrum. The most anterior part, the protocerebrum, can be further subdivided into a more lateral region bearing, for example, the optic lobes (archicerebrum) and a median region that includes, for example, the *pars intercerebralis *(prosocerebrum). Most authors think that the archicerebrum represents the tip of the neuraxis [1,5-8] but this has been disputed [9-11]. So far, it is unclear how the arthropod and annelid brain parts are related, if at all, and how they would align along the anterior-posterior axis [7,8,12,13]. In order to molecularly reassess this long-standing question, we have compared the expression of the anterior regionalisation genes *six3 *and *otx *during the development of annelid, arthropod and onychophoran brains.

## Results and discussion

**Figure 2:**
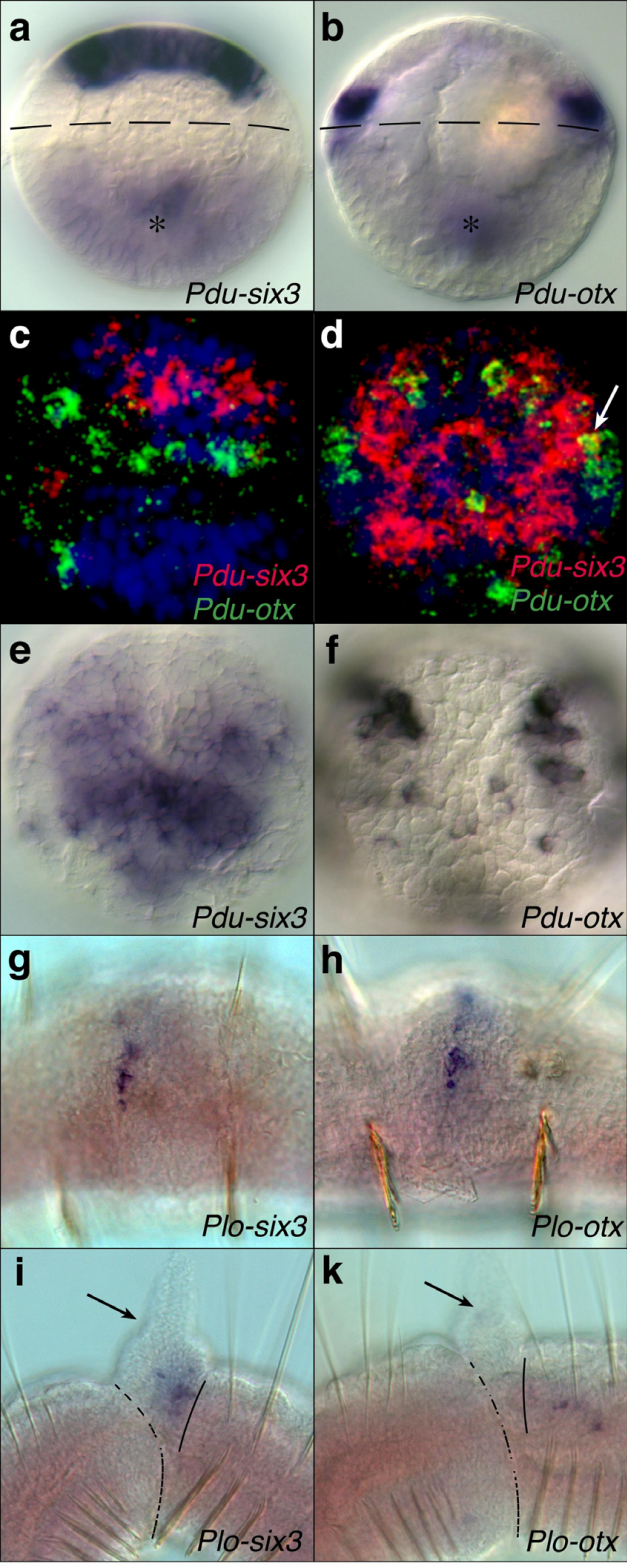
**Expression of annelid *otx *and *six3 *genes**. In the polychaete annelids *Platynereis *(a-f) and *Pristina *(g-k), *six3 *orthologues (a, c-e, g, i) are expressed anterior of *otx *orthologues (b-d, f, h, k). Single (a, b, e-k) or two-colour (c, d) whole-mount *in situ *hybridisations. Twenty-four hours (a-d) or 48 h (e, f) old *Platynereis *larvae. *Pristina *early (g, h) and late (i, k) fission stage. Asterisks in (a, b) point out stomodaeal expression (out of focus). Dashed line: Prototroch ciliary band. (c,d) Blue: nuclear DAPI stain. (i, k) Dotted line: Boundary of two forming worms dividing by fission; continuous line: *Plo-six3*/*Plo-otx *expression boundary. Arrows: Tentacles protruding dorsally from the anterior tip of the forming worm.

To elucidate head regionalisation in annelids (Figure 1b), we screened candidate genes for broad regional expression in the larval episphere and, at later developmental stages, in the prostomium. Previous studies identified molecular markers for sub-regions of the episphere and prostomium (for example, *Pdu-rx*, *Pdu-nk2.1*, *Pdu-pax6*) [14], for the equatorial ciliary girdle and mouth region giving rise to the non-metameric peristomium (*Pdu-otx*) [15,16], and for the posteriorly adjacent larval segment giving rise to the segmented trunk neuroectoderm (*gbx *[15] and *hox *[17]; Figure 1b). In order to identify a broad regionalisation marker for the anterior-most prostomium, we tested *six3*, because in vertebrates the spatially restricted expression of this gene demarcates the most anterior neural plate region [18] and is required for the formation of anterior structures [19]. *six3 *also demarcates the anterior body section of the enteropneust *Saccoglossus *[20] (Figure 1c) and of the sea urchin *Strongylocentrotus purpuratus *larvae [21], consistent with a conserved role in the specification of the front end of the body. In the marine annelid *Platynereis dumerilii *(Polychaeta, Phyllodocida), *Pdu-six3 *(Additional file 1: Supplementary Figure 1a) indeed proved to be a specific marker for almost the entire episphere, expressed at early (Figure 2a, c, d) and late larval stages (Figure 2e and Additional file 1: Supplementary Figure 2a, c). None of more than 100 other transcription factors tested showed a similarly broad and contiguous episphere-specific expression ([22] and data not shown). The broad apical domain of *Pdu-six3 *expression (Figure 2a, c, d) includes the anlagen of the antennae and palpae and is surrounded by the ring-like peristomial expression of *Pdu-otx *[16] (Figure 2b-d, Additional file 1: Supplementary Figure 2b, l), which covers equatorial/peristomial larval regions and overlaps with *six3 *in the periphery of the episphere (Figure 2d-f). The developing prostomium thus includes *six3+ *and *six3+/otx+ *co-expressing parts, while the peristomium expresses *otx *only (Figure 1b). Both *six3+ *and *otx+ *cells include neural progenitors and differentiating neurons as evidenced by co-expression with differentiation markers at 48 hpf (data not shown). As the positions of the mouth and eyes have often been used as landmarks to align the annelid and arthropod body regions, we also tried to affiliate the origin of these structures to the *six3+ *or *otx+ *regions. In *Platynereis*, *Pdu-six3 *is expressed in the stomodaeal roof (Additional file 1: Supplemental Figure 2 a, c), while the stomodaeal *Pdu-otx *expression starts broadly and becomes more restricted to single cells during later stages (Additional file 1: Supplementary Figure 2b, d). Thus, the stomodaeum is of mixed quality, but has its opening clearly surrounded by the *otx*+ peristomial region (Additional file 1: Supplementary Figure 2a, b, yellow arrowheads). At 24 hpf, the *Pdu-tryptophane-2,3-dioxygenase*-expressing rhabdomeric larval eyes express *Pdu-otx *(Additional file 1: Supplementary Figure 2l) but not *Pdu-six3 *(not shown). While the early *Pdu-six3+ *region is almost devoid of *Pdu-otx *expression, both genes overlap more broadly at later larval stages (Figure 2a-d, Additional file 1: Supplementary Figure 2c, d and data not shown) in brain regions that include the *Pdu-r-opsin*+ adult eyes [23] (Additional file 1: Supplementary Figure 2 l, m and data not shown). Thus, *otx *expression is shared by all eyes in *Platynereis *(as it is in *Drosophila*), while only a subset expresses additional *six3*, for example the *Platynereis *adult eyes (similar to the *Drosophila *compound eyes that express and require *six3/optix *[24]).

To obtain independent evidence that *six3 *plays a conserved role in outlining the most anterior head region in annelids, we cloned and investigated the expression of *otx *and *six3 *orthologs (Additional file 1: Supplementary Figure 1) in the oligochaete annelid *Pristina longiseta *that asexually reproduces by fission into chains of individuals that each regenerate a full anterior-posterior axis [25]. During early fission, both genes are expressed in stripes at the putative anterior part of the newly forming head in the middle of a segment (Figure 2g, h). At this stage, we were technically not able to resolve whether *Plo-six3 *lies anterior of *Plo-otx*. However, in later stages, using the developing antennae for spatial reference, we indeed observed a single patch of *Plo-six3 *expressing cells at the tip of a newly forming individual (Figure 2i), in front of *otx *expressing cells [26] (Figure 2k).

**Figure 3:**
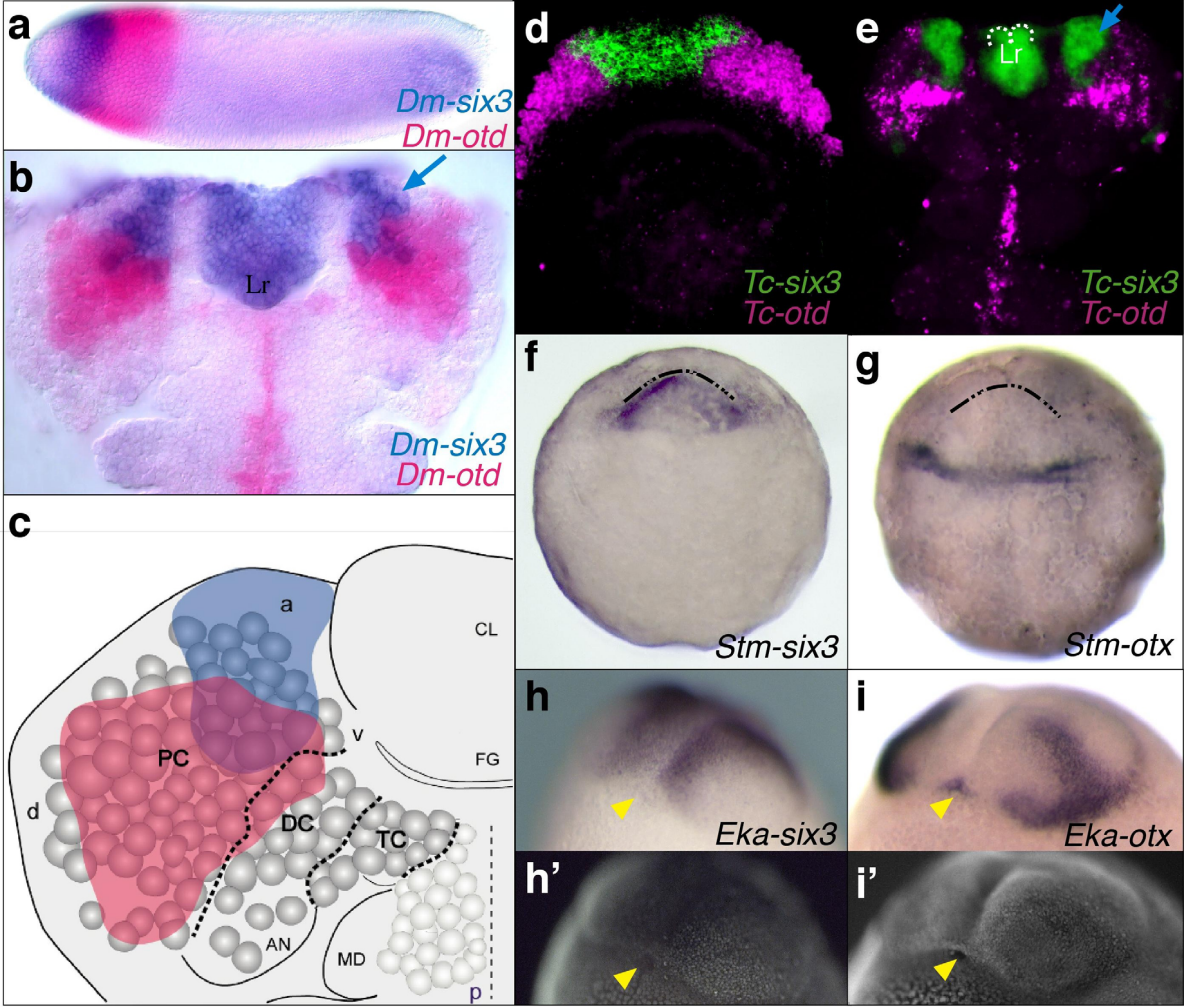
**Expression of insect, centipede and onychophoran *six3 *and *otx *genes**. In the fly *Drosophila *(a-c), the beetle *Tribolium *(d, e), the centipede *Strigamia *(f, g), the onychophoran *Euperipatoides *(h-i'), *six3*/*optix *orthologues (a-f, h) are expressed in an anterior-median location, while *otx*/*orthodenticle *orthologues (a-e, g, i) are expressed more posterior-laterally. Single (f-i') or two-colour (a, b, d, e) whole-mount *in situ *hybridisations. (a, b) *Drosophila *stage 6 (a) and 11 (b). (c) Schematics of *six3 *(blue) and *otx *(red) neuroectodermal expression in the left head hemisphere of a stage 11 *Drosophila*; expression of both genes is also detected in the underlying brain neuroblasts [36]. (d, e) *Tribolium *germ rudiment (d) and early elongating germband (e) stages. (f, g) *Strigamia *early segmentation stages. (h-i') *Euperipatoides *mid-segmentation stages. (h', i'): nuclear SYBRGreen stain of embryos in (h, i) for better visualization of the mouth opening. Dotted line in (e): Anterior labral border. Blue arrows in (b, e): *six3+ *neuroblasts. Dashed/dotted lines in (f, g): anterior germband margin. Yellow arrowheads in (h-i'): mouth opening. Abbreviations: a = anterior, AN = antennal segment, CL = clypeolabrum, d = dorsal, DC = deutocerebrum, FG = foregut, Lr = labrum, MD = mandibular segment, p = posterior, PC = protocerebrum, TC = tritocerebrum, v = ventral. Thin dashed line in (c): midline; thick dotted lines in (c): posterior borders of the protocerebrum, deuterocerebrum and tritocerebrum. (a): Lateral view. (b-g): Ventral views. (h-i'): Ventro-lateral views. All embryos with anterior to top except a: anterior to left.

We next tested whether a similar sequence of *six3*+ and *otx+ *regions also hallmarks the anterior end of the arthropod body (Figure 3). In the fly *Drosophila*, we found that *optix/six3 *indeed lies anterior of, and partly overlaps with, *orthodenticle*/*otx *expression at stage 6 (late blastoderm) and stage 11 (elongated germ band) (Figure 3a-c). However, since anterior-posterior patterning in *Drosophila *is known as being evolutionarily modified, we studied the beetle *Tribolium castaneum *where an *otx *gene ortholog forms part of a more ancestral anterior patterning system [27]. The expression of *Tc-six3 *(Additional file 1: Supplementary Figure 1a) demarcates a region at the tip of the germ rudiment [28], anteriorly adjacent to the expression region of *Tc-otd1 *(Figure 3d), which is the only beetle *otx *paralog expressed at early stages [29]. At the elongated germband stage, the *Tc-six3 *(Figure 3e) and *Drosophila six3 *(Figure 3b, c) expression regions are very similar and remain located at the anterior-median edge of the germband, including the labrum (Figure 3b, e), anterior brain neuroectoderm (Figure 3b, e) and corresponding neuroblasts (Figure 3c) [28] and is later also found in the developing stomodaeal roof (not shown). This result suggests that the role of *six3 *as a regional specification gene for the formation of the most anterior head and brain region, as shown in *Drosophila *and vertebrates, is conserved throughout Bilateria [19,30]. To validate evolutionary conservation of the anterior *six3 *region in other panarthropods, we isolated the *six3 *and *otx *orthologues (Additional file 1: Supplementary Figure 1) from the centipede *Strigamia maritima *(*Stm-six3*, *Stm-otx*) and from the velvet worm *Euperipatoides kanangrensis *(*Eka-six3*, *Eka-otx*) and for both species found *six3 *expressed in an anterior-median region at the tip of the germband and at later stages (Figure 3f, h and Additional file 1: Supplementary Figure 2e, g, i), while *otx *is mostly confined to more posterior and lateral coordinates (Figure 3g,i and Additional file 1: Supplementary Figure 2f, h, k). In *Euperipatoides*, the *Eka-six3 *domain includes the antennal anlagen, while the eye anlagen, as in other panarthropods, lie within the more lateral *Eka-otx+ *domain (Figure 3h-i', Additional file 1: Supplementary Figure 2i, k) [31,32]. As in *Platynereis *and *Drosophila *(Figure 3b), the mouth opening lies within a ventral patch of *otx *expressing cells (Figure 3i, i', yellow arrowheads). At late *Strigamia *stages, the mouth opening is broadly surrounded by *six3 *expression, but also expresses *otx *at the posterior border (Additional file 1: Supplementary Figure 2g, h). For *Euperipatoides *and *Strigamia*, the embryonic origin of the cells giving rise to the mouth is unclear.

**Figure 4:**
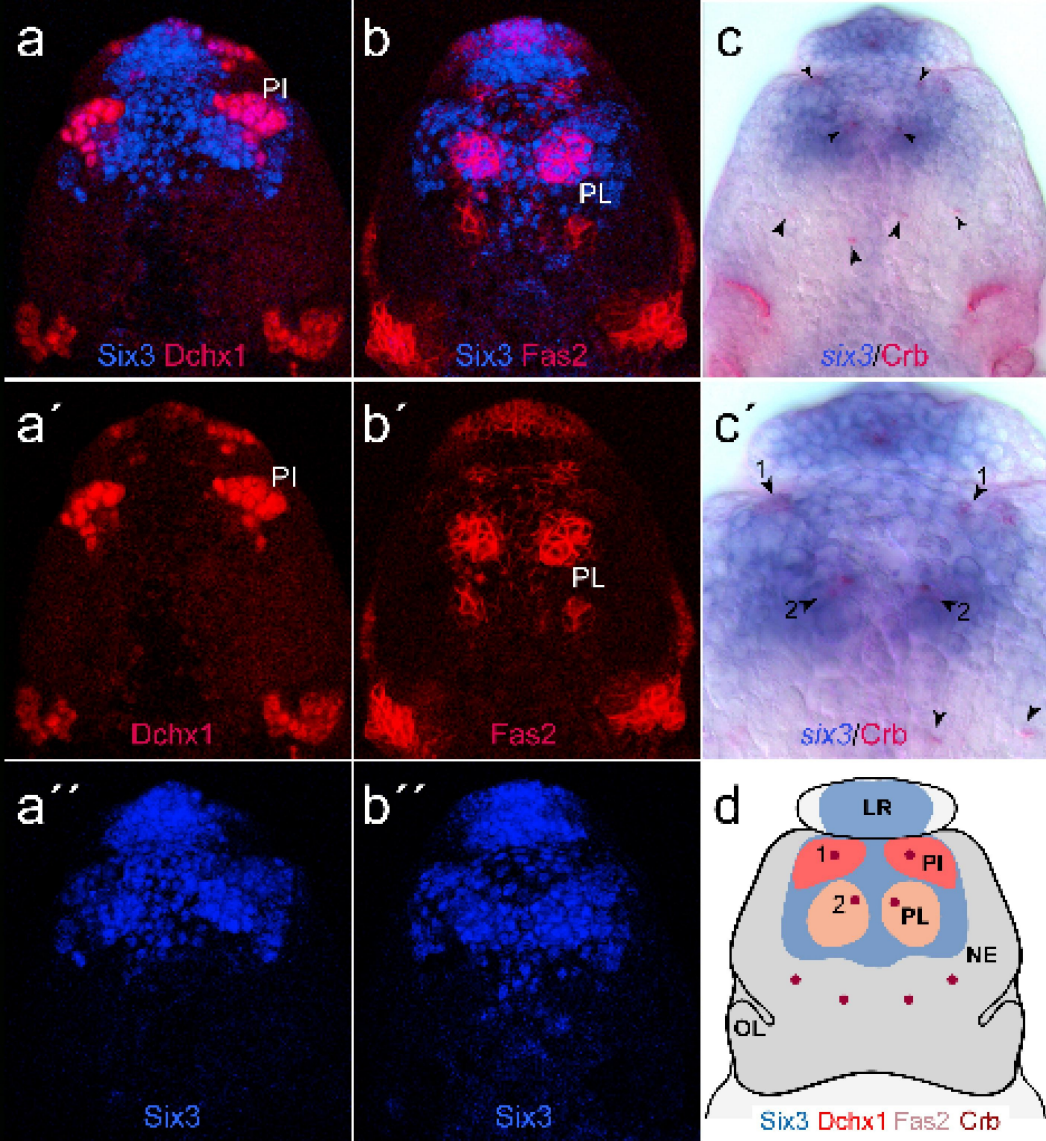
**The *Drosophila six3*/*opti*x-expressing region includes neurosecretory centres**. The neuroectodermal domains of the *Drosophila *neurosecretory *pars intercerebralis *(PI) and *pars lateralis *(PL) lie within the *six3*/*optix*-expressing region. (a, a', a'') Six3/Dchx1 protein expression. Six3 is detected in the neuroectoderm of the developing PI, as is specifically indicated by the expression of Dchx1. (b, b', b'') Six3/Fas2 protein expression. *Six3 *is additionally found to be expressed in the neuroectodermal placode of the developing PL, as is indicated by the strong expression of Fas2 [35]. (c, c') *six3 *mRNA/Crumbs protein expression. (c') Higher magnification of the *six3*-expressing head region. Black arrowheads in (c') depict invaginating placodal cells of the PI (1) and PL (2) as visualized by apically concentrated localisation of the Crumbs protein [35]; as is indicated by the red dots in (d). (d) Schematic summary of the expression of Six3, Dchx1, Fas2, and Crumbs in the anterior-dorsal head ectoderm, including the neuroectodermal placodes of the PI and PL, as is depicted by the colour code. LR = labrum; NE = neuroectoderm; OL = optic lobe anlagen; PI = pars intercerebralis; PL = pars lateralis.

What is the fate of the *six3*+ region in the diverse groups? In vertebrates, one prominent site of *six3 *activity is the developing hypothalamus [18,33]. Since in *Platynereis*, *Pdu-six3 *expression broadly covers the medial brain anlagen, it includes a large part of the early differentiating neurosecretory cells recently identified in the 48 hpf *Platynereis *brain anlage [14] (Additional file 1: Supplementary Figure 2c and data not shown). In insects, the neurosecretory *pars intercerebralis *and *pars lateralis *also originate from an anterior-median head position suggesting their origin from a *six3*-expressing region [34,35]. To validate this, we mapped *six3/optix *expression in the *Drosophila *head ectoderm and in brain neuroblasts (Figure 3b, c and Figure 4) [36]. We indeed found that the Six3+ dorsal brain region includes the developing Dchx1+ *pars intercerebralis *(Figure 4a-a'', d) and the Fas2+ *pars lateralis *(Figure 4b-b'', d), both also positive for the invaginating placode marker Crumbs (Figure 4c, c', d) [35]. Thus, the anlagen for the neurosecretory *pars intercerebralis *and *pars lateralis *lie within the *six3+ *region (Figure 3).

## Conclusions

Our comparative expression data shows that the developing annelid, arthropod and onychophoran heads comprise an anterior-most *six3+ *region and a more posterior *otx+ *region. Both regions are overlapping to a variable degree but show a clear anterior-to-posterior sequence, allowing cross-phylum alignment of head regions. In arthropods, the *six3+ *and *otx+ *head regions give rise to the protocerebrum and to the eyes (Figure 1a). In annelids, the *six3+*and *otx+ *regions cover the developing prostomium and the peristomium, from which the cerebral ganglia and eyes (and chemosensory appendages) develop (Figure 1b), but the *six3/otx*-based molecular subdivision does not fully match the morphological partition. While neuroectodermal *six3 *is restricted to the larval episphere and thus to the prostomium, the more posterior/equatorial *otx *expression covers the peristomium but also part of the prostomium where it overlaps with *six3*. Our data thus align annelid cerebral ganglia with arthropod protocerebrum (that is, the most anterior part of the arthropod cerebral ganglia, see "Background").

Many authors have argued that the most anterior structures in the arthropod brain are the anterior-lateral regions mainly consisting of the optic lobe [1,5-8]. These ocular regions largely coincide with the *otx+ *region (Figure 1a). Yet, the clear anterior location of the *six3*+ region in the early embryos of diverse arthropods, together with the role of *six3 *in defining the most anterior structures in other phyla, strongly suggest that it is this median *six3*+ region, giving rise to the neurosecretory *pars intercerebralis *and *pars lateralis *that represents the most anterior extreme of the arthropod brain (arrow in Figure 1a) and corresponds to the neurosecretory brain parts in annelids. This has hitherto been a minority view [9-11]. As the terms "archicerebrum" and "prosocerebrum" are tightly connected with the Articulata theory - unsupported by almost all molecular phylogenies - and have been inconsistently used to include different brain regions, we suggest abandoning these terms. Instead, our comparative studies reveal the existence of a conserved, ancient neurosecretory brain part at the tip of the neuraxis (Figure 1). It is followed by a more posterior part of the head (and brain) anlage expressing *otx *that often exhibits an early ring or arc-like pattern [29,37,38], consistent with the radial head hypothesis [39], and includes the eye anlagen (Figure 1). In the animals investigated, the position of the mouth opening is not reliably connected to the *six3 *or *otx *region: while it comes to lie within the *otx *region of *Platynereis *and onychophorans, its origin in arthropods is unclear. The fact that the annelid and onychophoran antennae develop from the *six3*+ region, in contrast to the arthropod antennae that develop posterior to the *otx*+ protocerebrum, is consistent with the previous assumption of homology between annelid and onychophoran antennae, but not with arthropod antennae [13]. The striking overall evolutionary conservation of a *six3*+ region in front of *otx*+ and *hox*+ regions in protostomes documented here (Figure 1), as well as in vertebrates and hemichordates, indicates that this anterior-posterior series may be universal to bilaterian animals.

## Methods

### Animal culture and collecting

*Platynereis *larvae obtained from an established breeding culture at EMBL, Heidelberg. *Strigamia maritima *eggs collected at Brora, Scotland (June 2006). Fly strains: Oregon R (wildtype). Female *Euperipatoides kanangrensis *Reid, 1996 were collected from decomposing logs of *Eucalyptus *trees in Kanangra Boyd National Park, NSW, Australia (33° 59'S 150° 08'E). Females were kept in containers with dampened sphagnum moss at 13°C and were fed crickets once every second week. Gravid females were relaxed and killed with ethyl acetate vapour from October to December in order to acquire embryos of correct stages. Embryos were dissected from the females in phosphate buffered saline (PBS) and, after removal of the egg membranes, fixed in 4% formaldehyde in PBS overnight at 4°C. Fixed embryos were dehydrated in a graded series of methanol (25, 50, 75% in PBS with 0.1% Tween-20 for 10 minutes each) and stored in 100% methanol at -20°C.

### Cloning of *six3*, *otx *and *tryptophane-2,3-dioxygenase *genes

All primers, PCR programs and template DNA source are given in Additional file 2. *Tc-six3 *gene was identified by *in silico *analysis of the *Tribolium *genome and amplified from a mixed stages (0 to 24h) cDNA library. Full length *Pdu-six3 *was isolated by screening a 48 h λ-ZAP phage library (provided by C. Heimann, Mainz). *Pdu-tryptophane-2,3-dioxygenase *gene was identified during a sequencing screen of a 48 h *Platynereis *EST library. Gene orthology was confirmed by using NCBI Protein BLAST, MUSCLE [40] multiple sequence alignments and CLUSTALX v.2 neighbour-joining phylogenetic analysis [41] for complete proteins.

### Database accession numbers

*Eka-otx*: EU347401, *Eka-six3*: EU347400, *Plo-otx*: EU330201; *Plo-six3*: EU330202; *Tc-six3*: AM922337; *Stm*-Six3: EU340980; *Stm*-otx: EU340979; *Pdu-six3*: FM210809; *Pdu-tryptophane-2,3-dioxygenase*: FN868644

### Whole-mount *in situ *hybridisation and immunohistochemistry

Established protocols were used for single- and two-colour fluorescent whole-mount *in situ *hybridisations of *Platynereis *and *Pristina *[42], *Euperipatoides *[43], *Strigamia *[44], *Drosophila *[45], and *Tribolium *[46]. A *Drosophila six3*/*optix *RNA probe was synthesized from EST clone LD05472 (Berkeley *Drosophila *Genome Project). Subsequent immunostainings were done using Vector Red (Vector Laboratories, Burlingame, CA, USA) or NBT/BCIP (Roche Diagnostics Penzberg, Germany)). Primary antibodies were: mouse anti-Crumbs (1:50; Developmental Studies Hybridoma Bank, DSHB), mouse anti-Fas2 (1:20; DSHB), rat anti-Orthodenticle [47] (1:1000, provided by T. Cook), guinea pig anti-Dchx1 antibody (1:1000; provided by T. Erclik), rabbit anti-Six3/Optix antibody (1:300; provided by F. Pignoni), alkaline phosphatase (AP)-coupled sheep anti-digoxygenin (1:1000, Roche). Secondary antibodies: AP-coupled donkey anti-rat, AP-coupled donkey anti-mouse, Cy5-coupled goat anti-rabbit (Dianova, Hamburg, Germany), Cy3-coupled goat anti-mouse (Dianova, , Hamburg, Germany). SYBRGreen (Invitrogen, San Diego, CA, USA) diluted 1:10.000.

## Abbreviations

AP: alkaline phosphatase; BCIP: 5-Bromo-4-Chloro-3'Indolyphosphate p-Toluidine; DSHB: Developmental Studies Hybridoma Bank EST: expressed sequence tags; otd: orthodenticle; NBT: Nitro-Blue Tetrazolium chloride; PBS: phosphate buffered saline; PCR: polymerase chain reaction; PI: pars intercerebralis; PL: Pars lateralis.

## Competing interests

The authors declare that they have no competing interests.

## Authors' contributions

PS analysed *Platynereis six3 *and *otx *expression, did multiple sequence alignments, conceived further experiments and wrote the paper. RU performed all *Drosophila *experiments. JE cloned and analysed *Euperipatoides six3 *and *otx *genes. NP performed *Tribolium *gene expression experiments. RK cloned and analysed *six3 *and *otx *genes in *Pristina*. CB cloned and analysed *Strigamia six3 *and *otx *genes. KG analysed co-expression of *Platynereis tryptophane-2,3-dioxygenase *and *otx *genes. MA and GB participated in the design of the study and the writing of the paper. DA designed the study, helped in writing the paper and cloned the *Platynereis six3 *gene.

## Supplementary Material

Additional file 1 **Supplementary figures and figure legends**. Steinmetz_Suppl_Figs.pdf contains two supplementary figures and legends showing multiple sequence alignments of *six3 *and *otx *genes, and supporting whole mount *in situ *hybridisation data of *Platynereis*, *Strigamia*, and *Euperipatoides *larva. Click here for file

Additional file 2 **Supplementary methods**. Steinmetz_SupplMethods.xls is an Excel Spreadsheet containing primer sequences, template source and PCR programs used to clone *six3 *and *otx *genes presented in the paper. Click here for file
