# Supplementary material for: Six3 demarcates the anterior-most developing brain region in bilaterian animals
Source: EvoDevo. 2010 Dec 29;1:14. doi: 10.1186/2041-9139-1-14 (PMC3025827; doi:10.1186/2041-9139-1-14)
Supplement: Additional file 1 — Supplementary figures and figure legends. Steinmetz_Suppl_Figs.pdf contains two supplementary figures and legends showing multiple sequence alignments of six3 and otx genes, and supporting whole mount in situ hybridisation data of Platynereis, Strigamia, and Euperipatoides larva. [file 2041-9139-1-14-S1.PDF]

Supplementary Figure 1

Partial protein multiple sequence alignment showing the homeobox and adjacent sequences of *six3* (a) and *otx* (b) orthologues in comparison with other closely related homeobox genes.

Only partial sequences available for *Plo-otx* and *Stm-otx*. Dot: conserved amino acid. Dash: Gap. Species abbreviations: *Bfl*: *Branchiostoma floridae*, *Dme*: *Drosophila melanogaster*, *Eka*: *Euperipatoides kanangrensis*, *Mmu*: *Mus musculus*, *Pca*: *Podocoryne carnea Pdu*: *Platynereis dumerilii*, *Plo*: *Pristina longiseta*, *Stm*: *Strigamia maritima*, *Tca*: *Tribolium castaneum*. Gene abbreviations: *optx*: *optix*, *so*: *sine oculis*, *otd*: *orthodenticle*. Gene accession numbers: *Bfl-otx*: AAC00193; *Dme-optx*: Q95RW8; *Dme-otd*: CAA41732; *Dme-six4/5*: NP_730566; *Dme-so*: Q27350; *Eka-otx*: ABY60730; *Eka-six3*: ABY60729; *Mmu-hesx1*: CAA56344; *Mmu-six1*: NP_033215; *Mmu-six2*: Q62232; *Mmu-six3*: Q62233; *Mmu-six4*: NP_035512; *Mmu-six5*: NP_035513; *Mmu-six6*: NP_035514; CAA56344; *Mmu-otx1*: NP_035153; *Mmu-otx2*: NP_659090; *Mmu-otx3*: NP_001020738; *Mmu*-vsx2: NP_001034513; *Pca-six1/2*: AAT11871; *Pca-six3/6*: AAT11872; *Pdu-otx*: CAC19028; *Pdu-six1/2*: CAC86663; *Pdu-six3*: FM210809; *Plo-otx*: ABY53615; *Plo-six3*: ABY50071; *Stm-otx*: ABY74501; *Stm-six3*: ABY74502; *Tca-otx*: NP_001034513; *Tca-six3*: CAP58434.

Supplementary Figure 2

Expression of *six3* and *otx* in different stages of *Platynereis*, *Strigamia*, and *Euperipatoides* and in relation to the larval eyes in *Platynereis.*

Single- (a-h) and two-colour (l-m) whole-mount *in situ* hybridisations of *six3* (a, c, e, g, i), *otx* (b, d, f, h, k-m), the *Platynereis* larval eye pigment cell marker gene *Pdu-tryptophane-2,3-dioxygenase* (l) and adult eye photoreceptor cell

marker *Pdu-r-opsin* (m). *Platynereis* larval stages: 24h (l), 36h (a, b) and 48h post-fertilization (c, d, m). *Strigamia* stages: 1st leg-bearing segment stage (e, f) and mid-germ band stage (g, h). *Euperipatoides* mid-segmentation stages (i, k). Yellow arrowheads in (a, b, g, h): mouth opening. sr: stomodaeal roof. (a-h): Ventral views. (i-m): Apical views; ventral to bottom.

a

```
                               homeobox
Mmu-six3     IWD-GEQKTHCFKERTRSLLREWYLQDPYPNPSKKRELAQATGLTPTQVGNWFKNRRQRDR-AA--AAKNRLQHQ
Mmu-six6     .................H...........................................-..--.......Q.
Plo-six3     VFERTQ.RS........N....S..R....S.GR..H..ES....................-..--.T...SRA.
Pdu-six3     .................N..............T............................-..--.....MNGH
Eka-six3     ................................T............................-..--.......QL
Stm-six3     ............R...................T............................-..--.......Q.
Tca-six3     ................................T............................-..--.....M.Q.
Dme-optx     ................................T......K....N................-..--.....I..S
Pca-six3/6   ........A........K....F.......S.....D..D..H..................-..--....KS.R.
Mmu-six1     ......ETSY....KS.GV.....AHN...S.RE.....E.....T...S...........-..--EAKERENTE
Mmu-six2     ......ETSY....KS..V.....AHN...S.RE.....E.....T...S...........-..--EAKERENSE
Pdu-six1/2   ......ETSY....KS.TV.....AHN...S.RE.....E.....T...S...........-..--EVKDSRDGP
Pca-six1/2   ......ETSY....KS.AV..D..TRN...S.RE.K..SEG...ST...S...........-..--EAKEREEES
Dme-so       ......ETSY....KS..V..D..SHN...S.RE..D..E.....T...S...........-..--EHKDGSTDK
Mmu-six4     ......ETVY....KS.NA.K.L.K.NR..S.AE..H..KI...SL...S...........NPS--ETQSKSESD
Mmu-six5     ......ETVY.....S.AA.KAC.RGNR..T.DE..R..TL...SL...S...........TGTGGGAPCKSESD
Dme-six4/5   ......ETVY....KS.NA.KDC..TNR..T.DE.KT..KK....L...S...........-T------------
```

b

```
                                                 homeobox
Mmu-otx2     YPATPRKQ----------RRERTTFTRAQLDVLEALFAKTRYPDIFMREEVALKINLPESRVQVWFKNRRAKCRQQQQQ-------------------
Mmu-otx1     ........----------.........S.................................................---------------------
Mmu-otx3     .GSQH...----------..S..A..AQ..EA..KT.Q..H...VV...RL.MCT....A............F.KK.RSLQKEQLQKQK---------
Bfl-otx      C.PP....----------..........................................................AG.AK-----------------
Pdu-otx      EA.N....----------................S..Q.......................................KA-------------------
Plo-otx      ...SS...----------.......S.S......T..H..........--------------------------------------------------
Eka-otx      -------.----------...................S..................F......................QQQ----------------
Stm-otx      -----------------------------------------------------------------..............-------------------
Tca-otd      AGVN....----------.............L..G.................V.......................L..QQ-----------------
Dme-otd      PGVNT...----------...................G......................................L..QQQSNSLSSSKNASGGGS
Mmu-Hesx1    FS.SETRSLKRELSWYRG..P..A..QN.VE...NV.RVNC..G.DI..DL.Q.L..E.D.I.I..Q.....MKRSRRE------------------
Mmu-vsx2     NQTKK..K----------..H..I..SY..EE..KA.NEAH...VYA..ML.M.TE...D.I....Q.....W.KREKC------------------

Mmu-otx2     --------------------------QQNGGQNKVRPAKKKSSPAREVSSESGTS--GQFSPPSSTSVPTIASSSAP--
Mmu-otx1     --------------------------..S.NGT.T..V......V..S.GSESS---...T..AVS.SASSS..ASSAS
Mmu-otx3     --------------------------EAE.SHGEGKVEAPA.DTQL.TEQPP.LP--SGDP.AELQLSLSEQ.A.ESAP
Bfl-otx      --------------------------PRPKKKSASP.PASTEEQ.PTSE.P.CSDTSVSST.VPVAVTIGNTNA.PTAA
Pdu-otx      ---------------------------.D.RNPAS..K.S..PT------------PGDVS.VNNNTSSDS.YKTVAP
Plo-otx      ------------------------------------------------------------------------------
Eka-otx      --------------------------....N...S..K.A.TP.PPTSRASPYK---PPSV.AVNSMT.PG--------
Stm-otx      --------------------------........S..K.P..P.PPAT.TSPPS.RDSPYK..V..PTAAVGLAPP.PS
Tca-otd      --------------------------NKSASRTTTS.T.V.A.K.SPAAAPRSVATPTGIPT..TSAS.PTVNIKKESP
Dme-otd      GNSCSSSSANSRSNSNNNGSSSNNNT.SS..N.SNKSSQ.QGNS--QS.QQG.G.--SGGNNSNNN.AAAA..AA.AVA
Mmu-Hesx1    ------------------------------------------------------------------------------
Mmu-vsx2     ----------------------------------------------------------WGR.SVMAEYGLYG.MVR----
```

Steinmetz et al.
Supplementary Figure 1

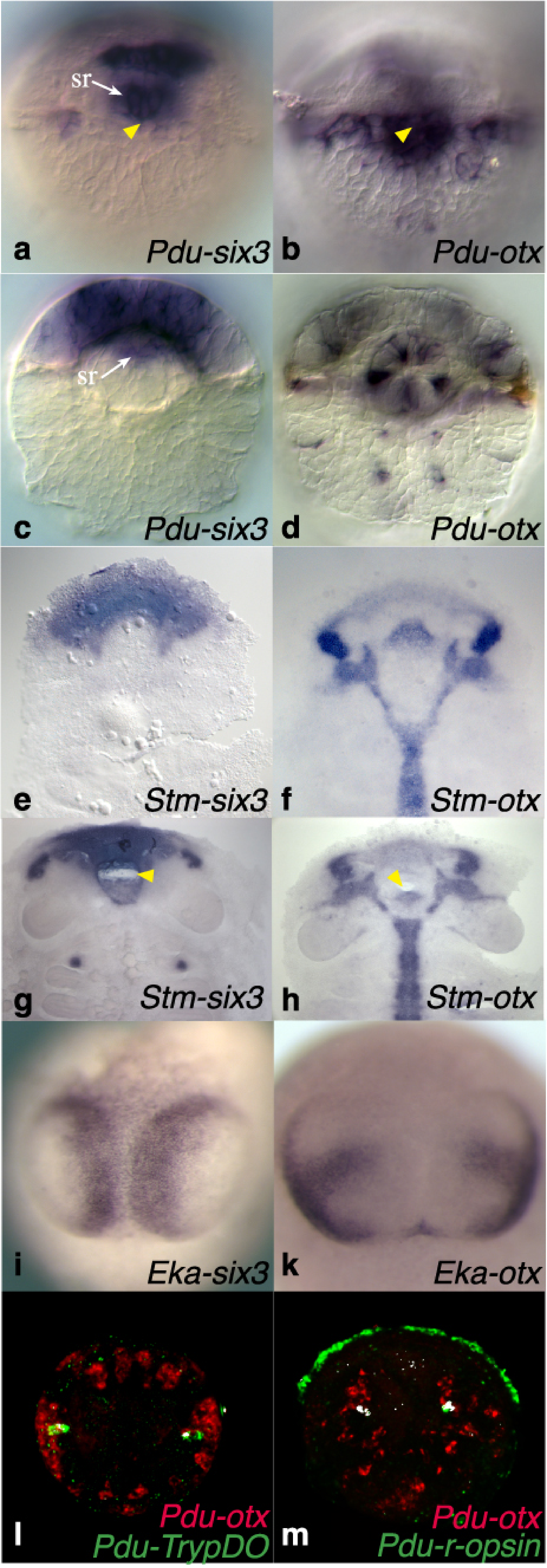

Steinmetz et al.
Supplementary Figure 2
